# Supplementary material for: Burden of chronic kidney disease in the general population and high-risk groups in South Asia: A systematic review and meta-analysis
Source: PLoS One. 2021 Oct 14;16(10):e0258494. doi: 10.1371/journal.pone.0258494 (PMC8516300; doi:10.1371/journal.pone.0258494)
Supplement: S1 Table — (PDF) [file pone.0258494.s002.pdf]

S1 Table. Search strategy

**PubMed**<br>("kidney diseases" [MeSH Terms] OR "kidney disease" OR "kidney failure" OR "kidney insufficiency" OR "kidney function" OR "kidney dysfunction" OR "renal disease" OR "renal failure" OR "renal insufficiency" OR "renal function" OR "renal dysfunction") AND (India OR Bangladesh OR Sri Lanka OR Nepal OR Bhutan OR Maldives OR Pakistan OR Afghanistan OR "South Asian Association for Regional Cooperation" OR SAARC OR South Asia) AND (Epidemiology OR prevalence [MeSH Terms] OR prevalence)
**SCOPUS**<br>TITLE-ABS-KEY ("kidney disease" OR "kidney failure" OR "kidney insufficiency" OR "kidney function" OR "kidney dysfunction" OR "renal disease" OR "renal failure" OR "renal insufficiency" OR "renal function" OR "renal dysfunction") AND TITLE-ABS-KEY (India OR Bangladesh OR Sri Lanka OR Nepal OR Bhutan OR Maldives OR Pakistan OR Afghanistan) AND TITLE-ABS-KEY (Epidemiology OR prevalence)
**Web of Science**<br>TOPIC: ("kidney disease" OR "kidney failure" OR "kidney insufficiency" OR "kidney function" OR "kidney dysfunction" OR "renal disease" OR "renal failure" OR "renal insufficiency" OR "renal function" OR "renal dysfunction") AND TOPIC: (India OR Bangladesh OR Sri Lanka OR Nepal OR Bhutan OR Maldives OR Pakistan OR Afghanistan) AND TOPIC: (Epidemiology OR prevalence)
