# Supplementary material for: Burden of chronic kidney disease in the general population and high-risk groups in South Asia: A systematic review and meta-analysis
Source: PLoS One. 2021 Oct 14;16(10):e0258494. doi: 10.1371/journal.pone.0258494 (PMC8516300; doi:10.1371/journal.pone.0258494)
Supplement: S2 Table — (PDF) [file pone.0258494.s003.pdf]

S2 Table. Quality assessment of the included studies using the Hoy et al. risk of bias assessment tool.

Study ID	External validity				Internal validity						Scores	
	Was the study's target population a close representative of the general population or, representative of the population in question	Was the sampling frame a true or close representation of the target population?	Was some form of random selection used to select the sample, OR was a census undertaken?	Was the likelihood of nonresponse bias minimal?	Were data collected directly from the subjects (as opposed to a proxy)?	Was an acceptable case definition used in the study?	Was the study instrument that measured the parameter of interest shown to have validity and reliability?	Was the same mode of data collection used for all subjects?	Was the length of the shortest prevalence period for the parameter of interest appropriate?	Were the numerator(s) and denominator(s) for the parameter of interest appropriate?	Total score	Methodological quality
Anand 2015[17]	1	1	1	1	1	1	1	1	1	1	10	High Quality
Anupama 2014[18]	1	1	1	0	1	1	1	1	1	1	9	High Quality
Farag 2020[19]	1	1	1	1	1	1	1	1	1	1	10	High Quality
Fatema 2013[20]	1	0	0	0	1	1	1	1	1	1	7	Moderate Quality
Feng 2018[21]	1	1	1	1	1	1	1	1	1	1	10	High Quality
Hasan, 2012[22]	0	0	0	0	1	1	1	1	1	1	6	Moderate Quality
Huda 2012[23]	1	0	1	0	1	1	1	1	0	1	7	Moderate Quality
Jayatilake 2013[24]	0	1	1	0	1	1	1	1	1	1	8	Moderate Quality

Jessani 2014[25]	1	1	1	0	1	1	1	1	1	1	9	High Quality
Khanam 2016[26]	0	0	1	0	1	1	1	1	1	1	7	Moderate Quality
Mahapatra 2016[27]	0	0	0	0	1	1	1	1	1	1	6	Moderate Quality
Mohanty 2020[28]	1	1	1	0	1	1	1	1	1	1	9	High Quality
O'callaghan 2019[29]	1	1	1	0	1	1	1	1	1	1	9	High Quality
Rajput 2017[30]	1	1	0	0	1	1	1	1	1	1	8	Moderate Quality
Ruwanpathirana 2019[31]	0	1	1	1	1	1	1	1	1	1	9	High Quality
Selected NCDs 2019[40]	1	1	1	1	1	1	1	1	1	1	10	High Quality
Sharma 2013[32]	0	0	0	0	1	1	1	1	1	1	6	Moderate Quality
Sharma 2010[33]	1	1	0	0	1	0	1	1	1	1	7	Moderate Quality
Singh 2009[35]	1	1	1	0	1	0	1	1	1	1	8	Moderate Quality
Singh 2013[34]	1	0	0	0	1	1	1	1	0	1	6	Moderate Quality
Tatapudi 2019[36]	0	1	1	1	1	1	1	1	1	1	9	High Quality
Trivedi 2016[37]	0	0	0	0	1	1	1	1	1	1	6	Moderate Quality
Varma 2010[39]	1	0	0	0	1	1	1	1	1	1	7	Moderate Quality
Varma 2011[38]	1	0	1	0	1	1	1	1	1	1	8	Moderate Quality
