# Supplementary material for: Burden of chronic kidney disease in the general population and high-risk groups in South Asia: A systematic review and meta-analysis
Source: PLoS One. 2021 Oct 14;16(10):e0258494. doi: 10.1371/journal.pone.0258494 (PMC8516300; doi:10.1371/journal.pone.0258494)
Supplement: S3 Table — (PDF) [file pone.0258494.s004.pdf]

S3 Table. Meta-regression analysis for the variance of the prevalence of chronic kidney disease

Variables (reference)	No of studies	Univariate analysis		
		**Coefficient (95% CI)**	**P-value**	**R^2**
Mean Age	15	0.003 (-0.005, 0.011)	0.429	3.6%
Mean BMI	6	-0.1 (-0.09, 0.07)	0.76	23.3%
Survey year	16	0.0007 (-0.008, 0.01)	0.847	8.3%
