# Supplementary figures and images for: Burden of chronic kidney disease in the general population and high-risk groups in South Asia: A systematic review and meta-analysis
Source: PLoS One. 2021 Oct 14;16(10):e0258494. doi: 10.1371/journal.pone.0258494 (PMC8516300; doi:10.1371/journal.pone.0258494)

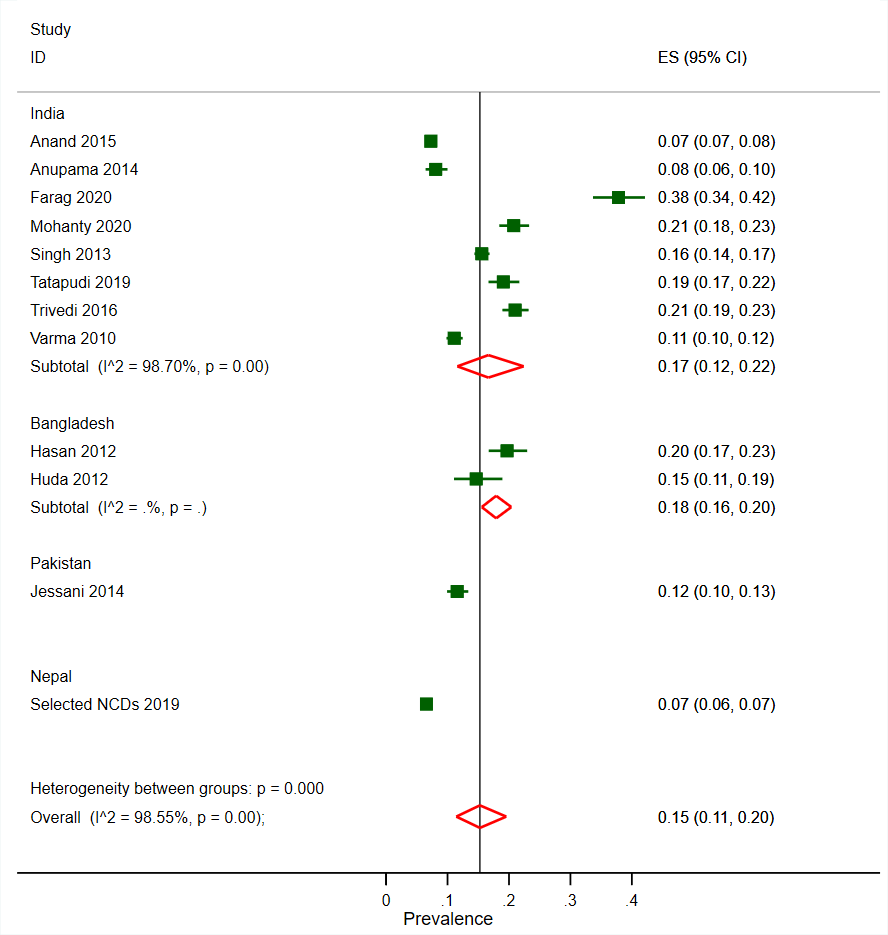

Supplement: S1 Fig — (TIF) [file pone.0258494.s005.tif]

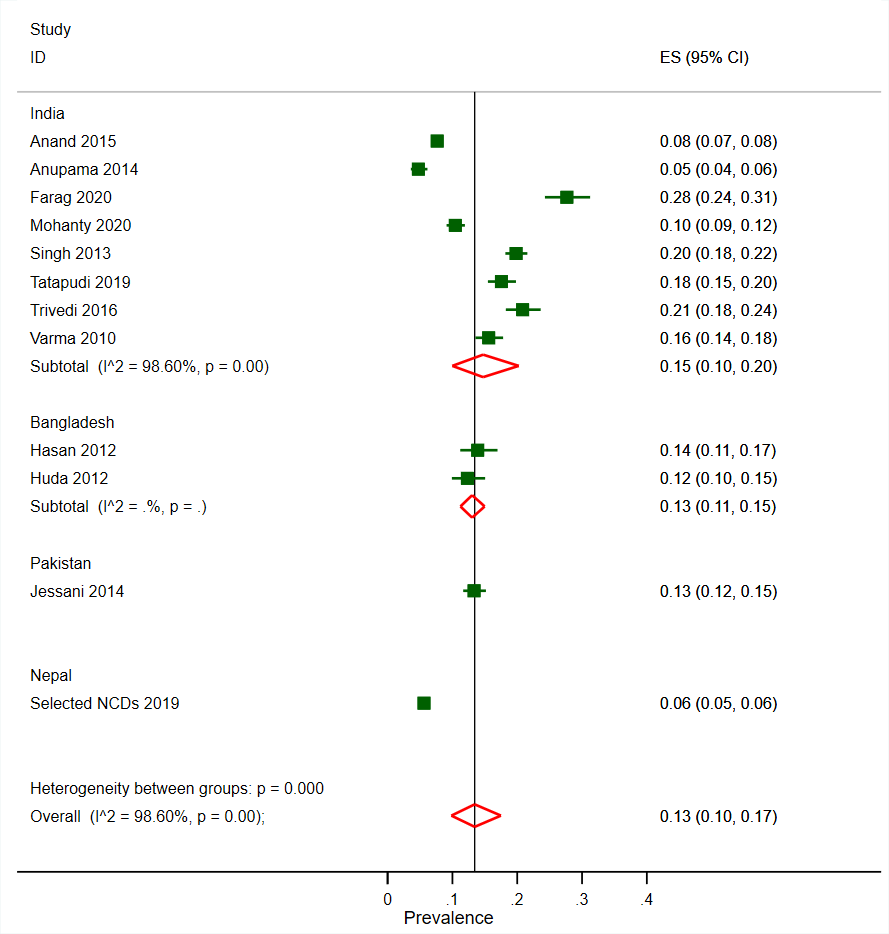

Supplement: S2 Fig — (TIF) [file pone.0258494.s006.tif]

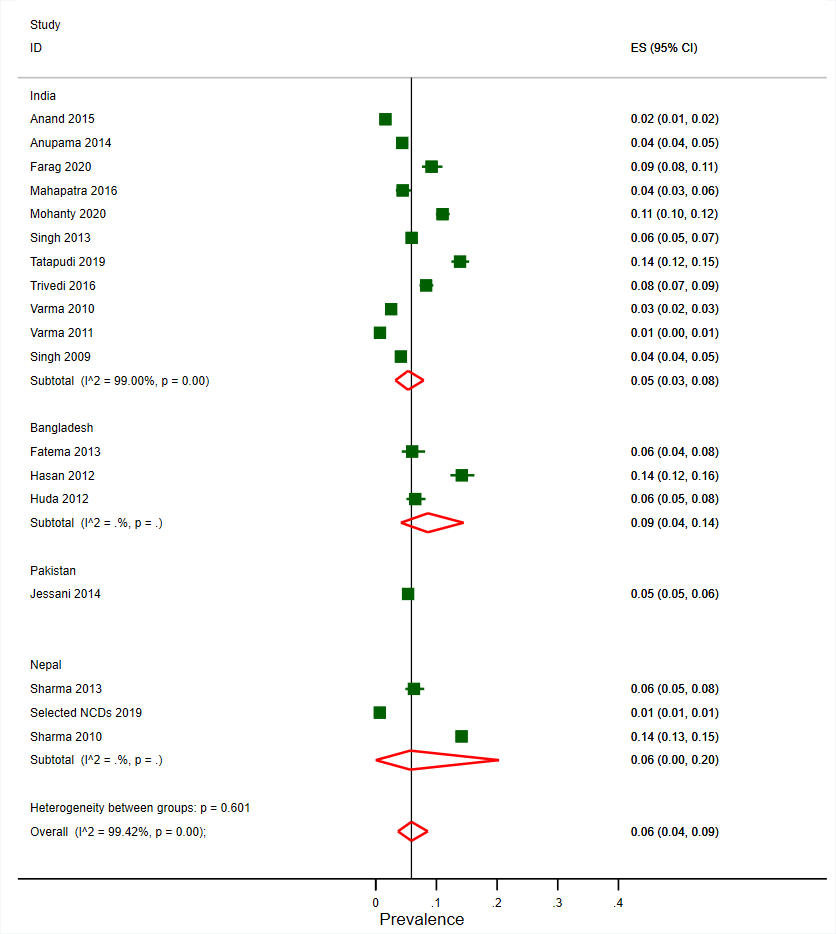

Supplement: S3 Fig — (TIF) [file pone.0258494.s007.tif]

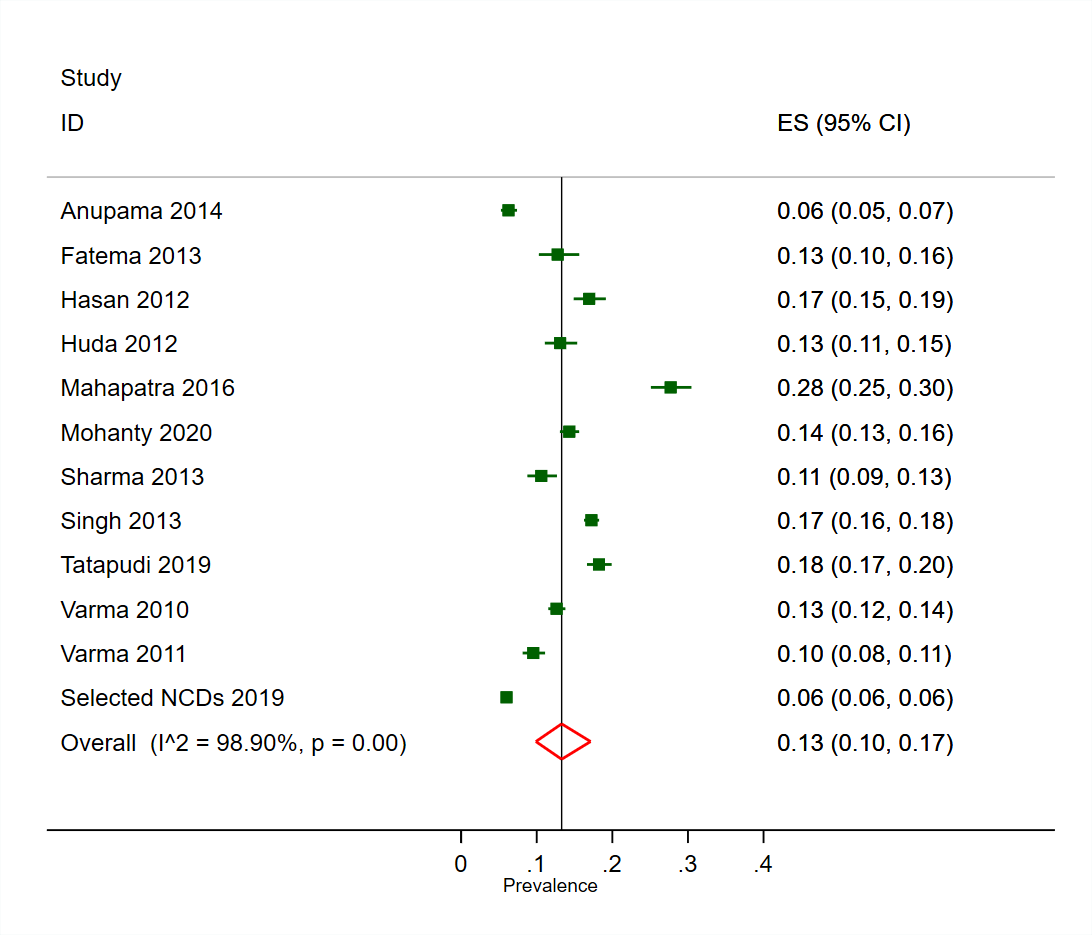

Supplement: S4 Fig — (TIF) [file pone.0258494.s008.tif]

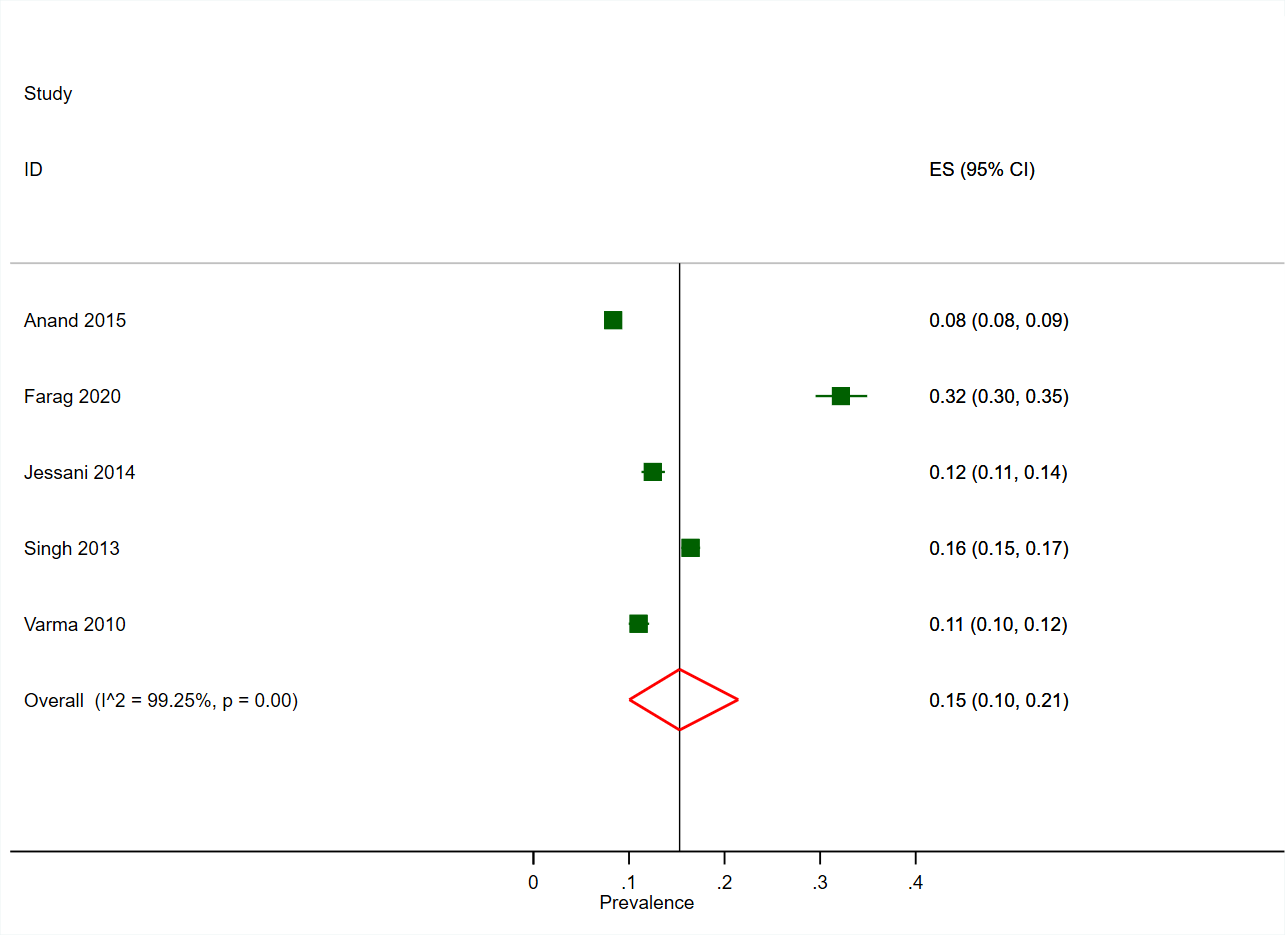

Supplement: S5 Fig — (TIF) [file pone.0258494.s009.tif]

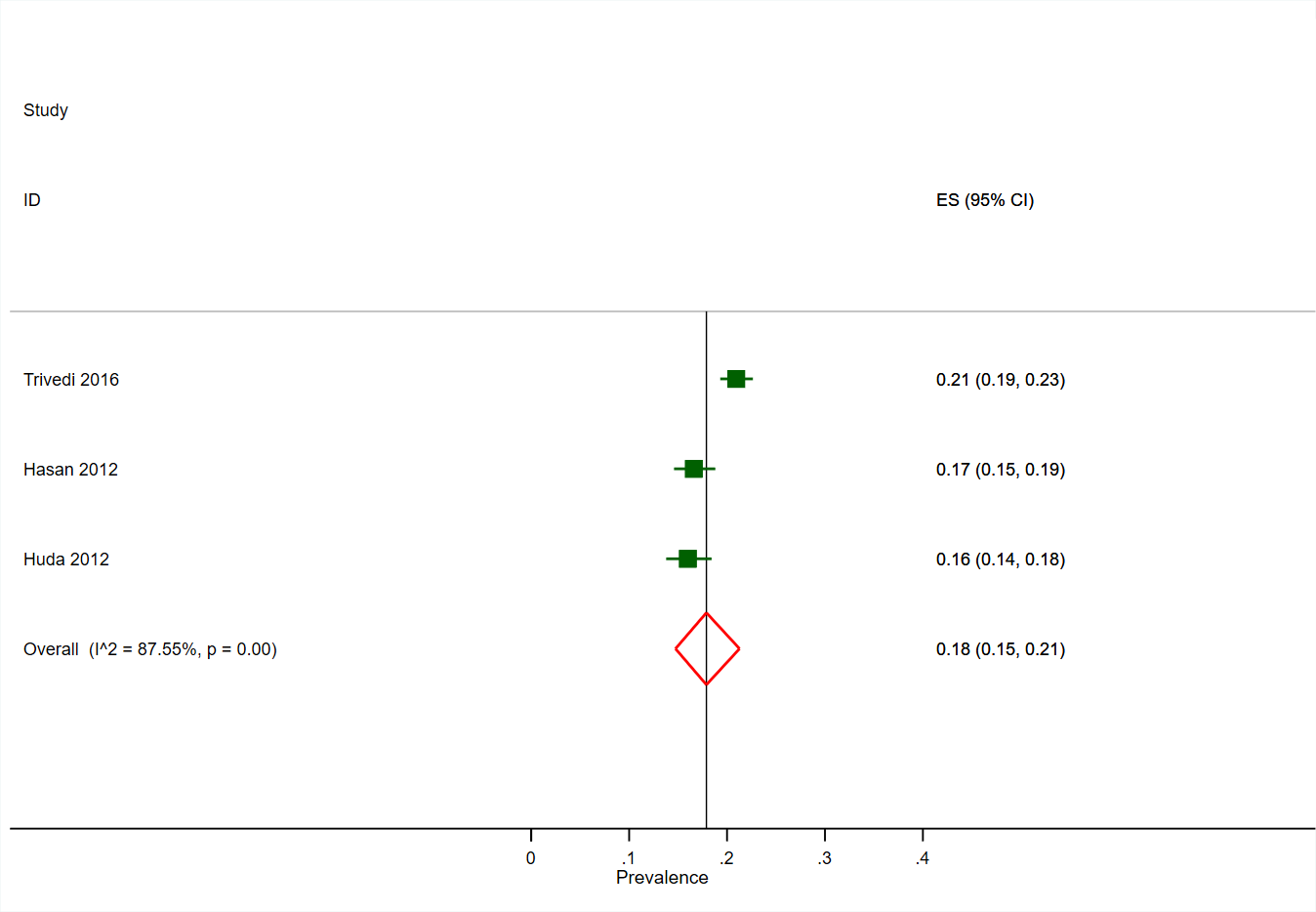

Supplement: S6 Fig — (TIF) [file pone.0258494.s010.tif]

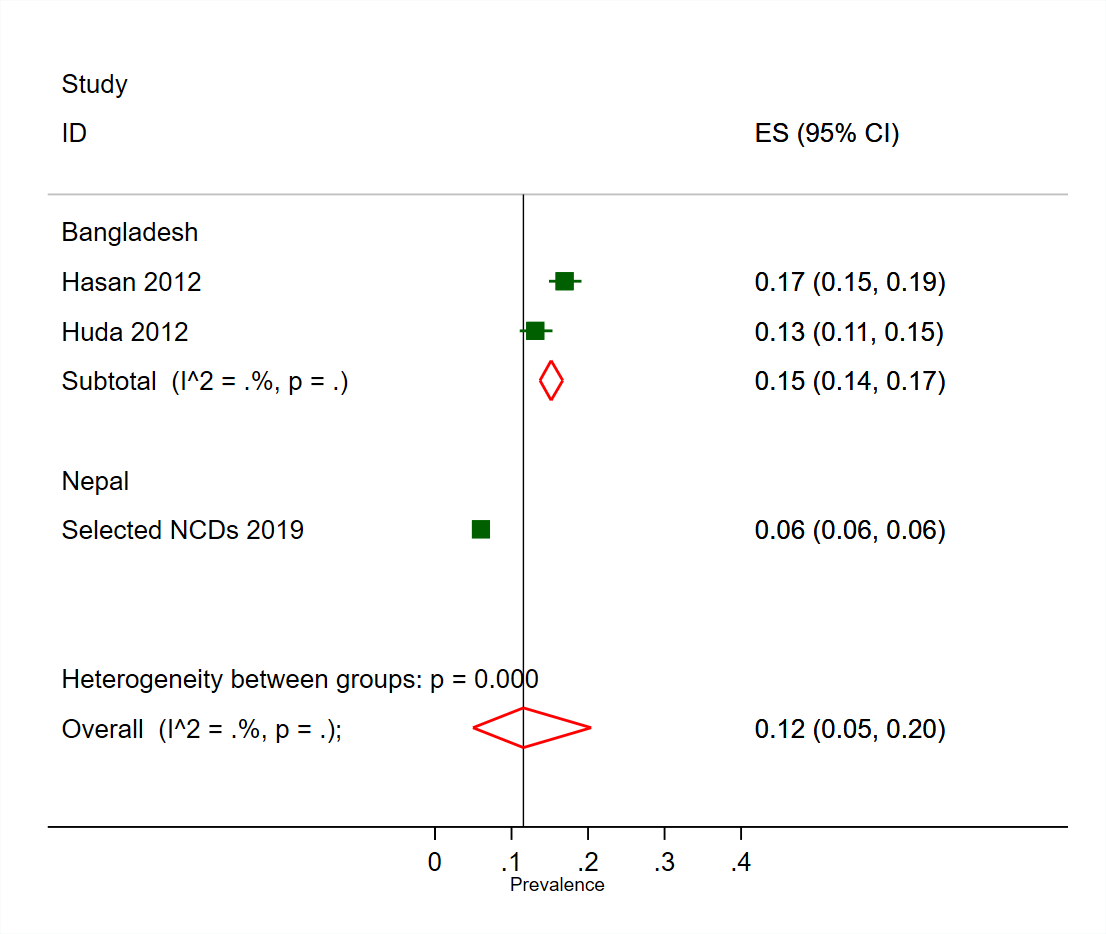

Supplement: S7 Fig — (TIF) [file pone.0258494.s011.tif]

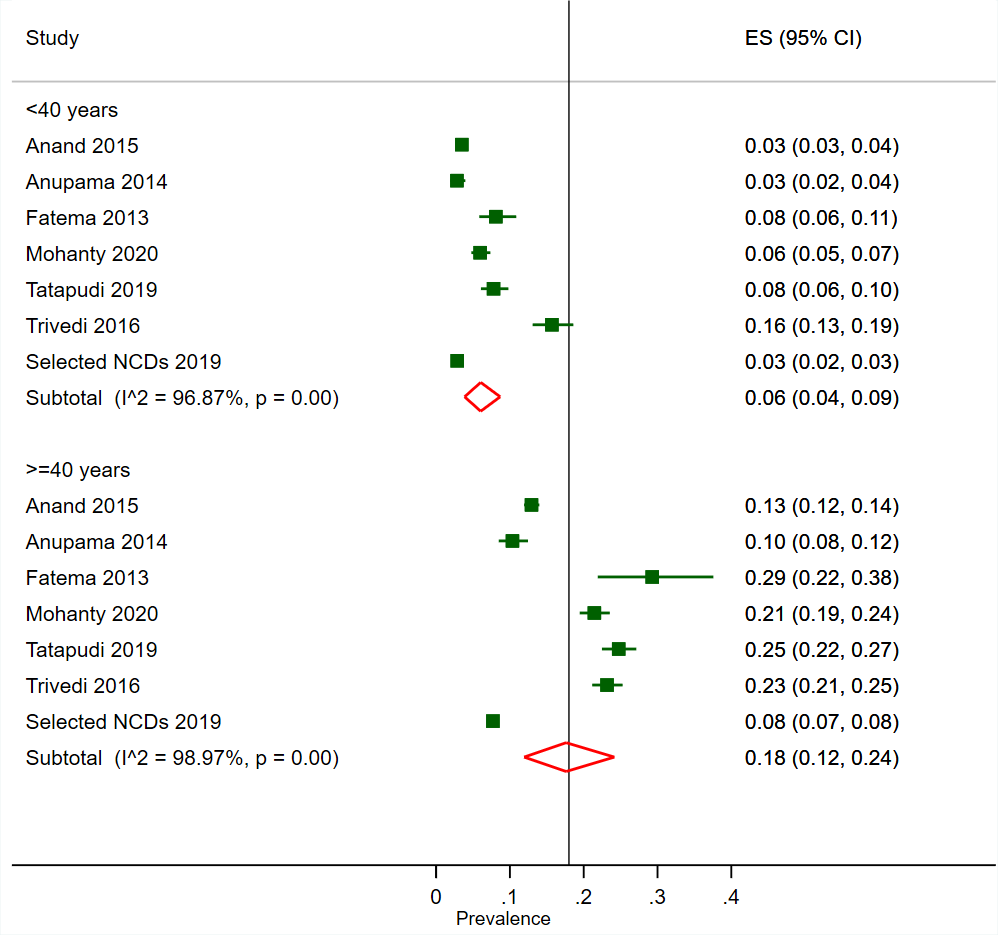

Supplement: S8 Fig — (TIF) [file pone.0258494.s012.tif]

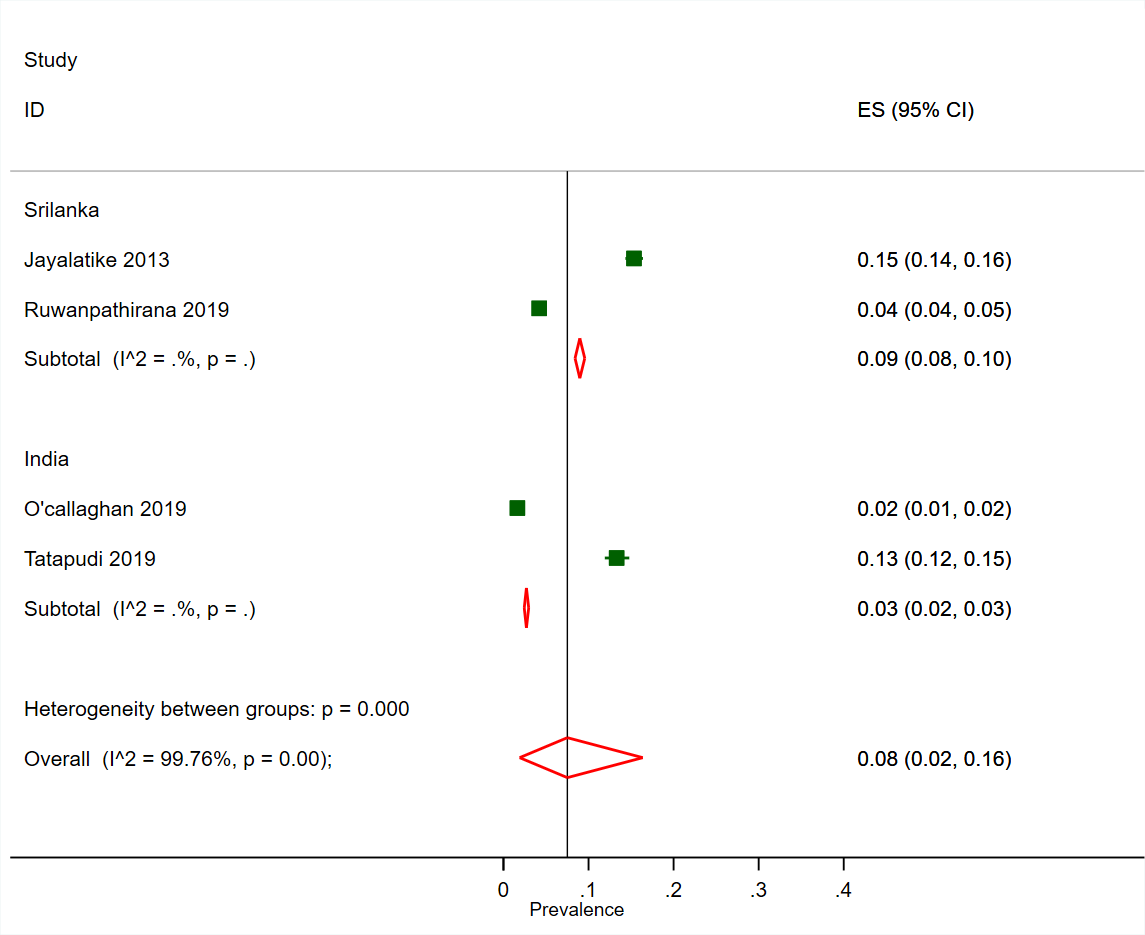

Supplement: S9 Fig — (TIF) [file pone.0258494.s013.tif]

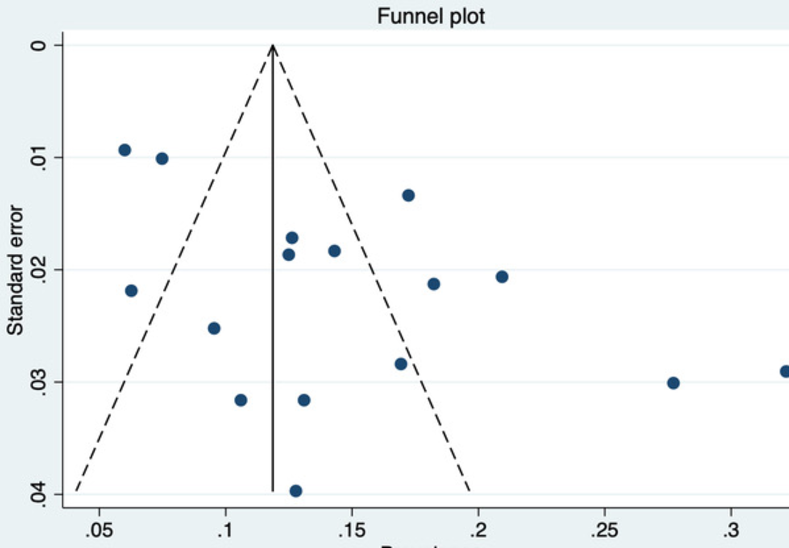

Supplement: S10 Fig — (TIF) [file pone.0258494.s014.tif]

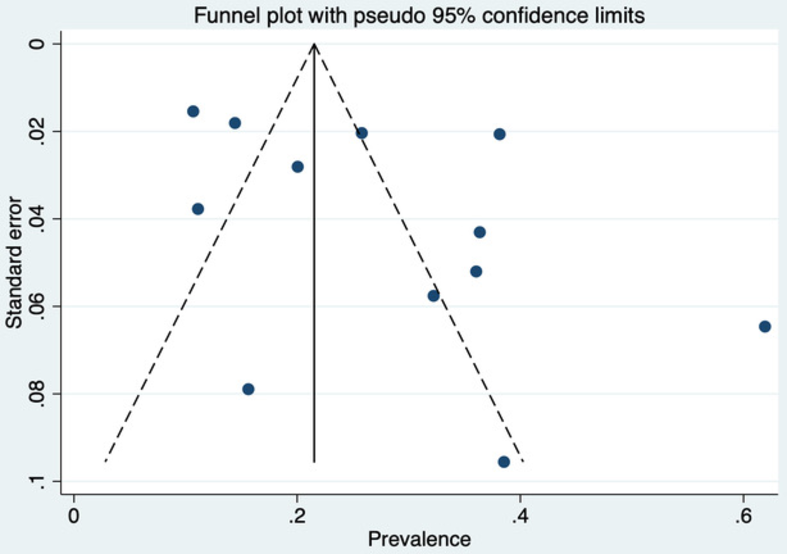

Supplement: S11 Fig — (TIF) [file pone.0258494.s015.tif]

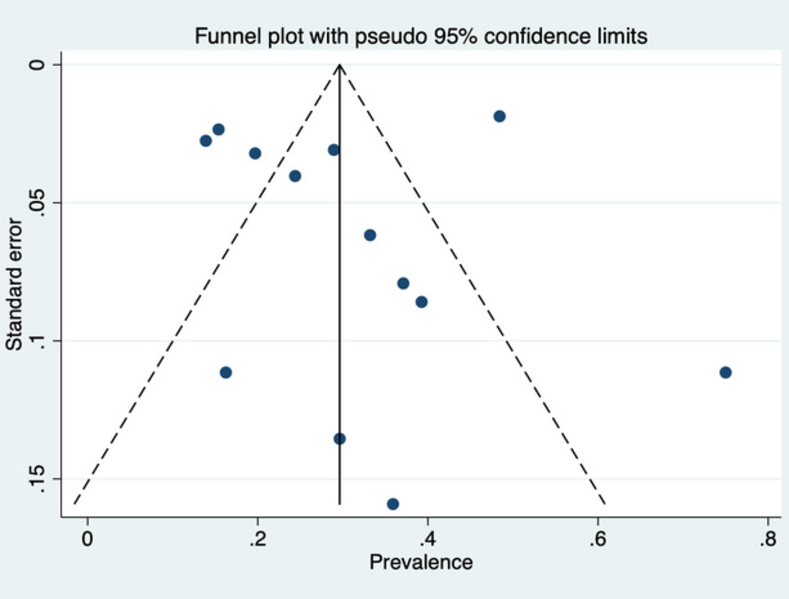

Supplement: S12 Fig — (TIF) [file pone.0258494.s016.tif]

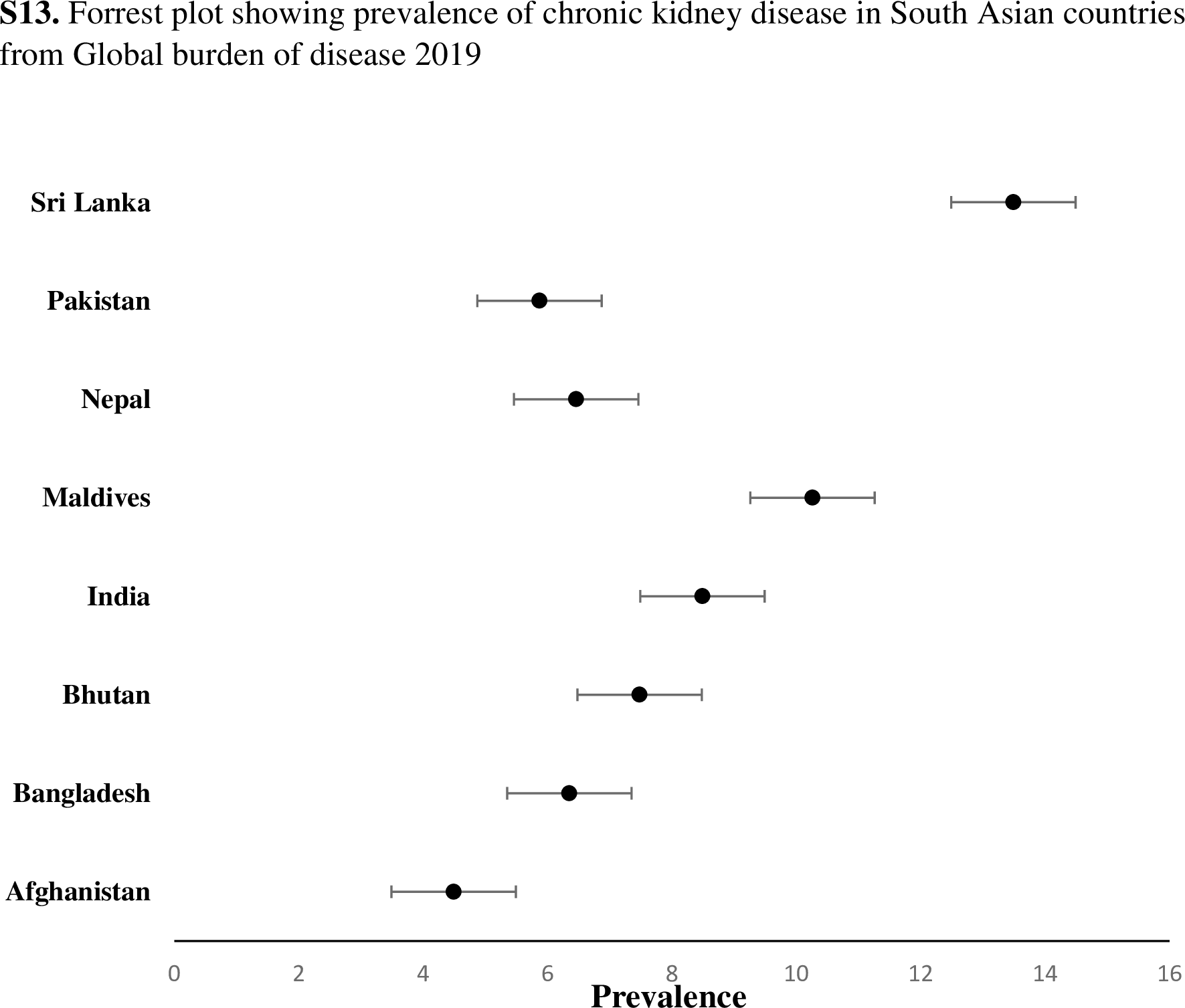

Supplement: S13 Fig — (TIF) [file pone.0258494.s017.tif]
